# Oligoprogression During First-Line Treatment of Metastatic Hormone-Sensitive Prostate Cancer: Comparative Outcomes of Metastasis-Directed Radiotherapy and Systemic Treatment Change

**DOI:** 10.3390/jcm15083067

**Published:** 2026-04-17

**Authors:** Murat Günaltılı, Murad Guliyev, Zeliha Birsin, Emir Çerme, Vali Aliyev, Hamza Abbasov, Selin Cebeci, Seda Jeral, Ebru Çiçek, Süheyla Atak, Halil Cumhur Yıldırım, Nebi Serkan Demirci, Fazilet Öner Dinçbaş, Özkan Alan

**Affiliations:** 1 Division of Medical Oncology, Department of Internal Medicine, Cerrahpaşa Faculty of Medicine, Istanbul University-Cerrahpaşa, Istanbul 34098, Turkey; drmuradguliyev@gmail.com (M.G.); zelihabirsin@gmail.com (Z.B.); emircrm34@gmail.com (E.Ç.); dktr.aliyev@gmail.com (V.A.); hamzaabbasov90@gmail.com (H.A.); sellcebeci@gmail.com (S.C.); sedajeral@gmail.com (S.J.); ebruhct@gmail.com (E.Ç.); suheylakeles93@gmail.com (S.A.); drserkannebi@yahoo.com (N.S.D.); ozkan.alan@hotmail.com (Ö.A.); 2 Department of Radiation Oncology, Cerrahpaşa Faculty of Medicine, Istanbul University-Cerrahpaşa, Istanbul 34098, Turkey; halilcumhur.yildirim@iuc.edu.tr (H.C.Y.); fazilet.dincbas@iuc.edu.tr (F.Ö.D.)

**Keywords:** metastasis-directed radiotherapy, metastatic hormone-sensitive prostate cancer, oligoprogression, prostate cancer, radiologic progression-free survival

## Abstract

**Background/Objectives:** We evaluated the clinical outcomes of two commonly used approaches for managing oligoprogression arising during first-line therapy for metastatic hormone-sensitive prostate cancer (mHSPC): metastasis-directed radiotherapy (RT) with continuation of the ongoing systemic regimen and immediate transition to another systemic treatment. **Methods:** A total of 81 patients with mHSPC who experienced radiologically confirmed oligoprogression during first-line systemic therapy were retrospectively evaluated. Oligoprogression was defined as progression involving three or fewer metastatic sites. Patients were categorized into an RT group (metastasis-directed RT with continuation of the same regimen) or a treatment-change group (immediate switch in systemic therapy without RT). Post-oligoprogression radiologic progression-free survival (rPFS) and overall survival (OS) were evaluated using Kaplan–Meier estimates and Cox proportional hazards models. **Results:** Thirty-one patients received metastasis-directed RT, whereas fifty underwent a change in systemic therapy. The median post-oligoprogression rPFS was 25.8 months (95% CI, 16.3–35.2) in the entire cohort and did not differ significantly between the treatment-change (26.8 months) and RT groups (22.7 months; *p* = 0.828). The median OS was 42.6 months overall, with comparable outcomes between the treatment-change (42.6 months) and RT groups (52.4 months; *p* = 0.452). **Conclusions:** In patients with mHSPC who developed oligoprogression during first-line systemic therapy, metastasis-directed RT with continuation of the same regimen and immediate change in systemic treatment were associated with comparable post-oligoprogression outcomes in our cohort. These findings suggest that both strategies may be feasible in selected patients. Prospective studies may help clarify which patients are more likely to benefit from each strategy.

## 1. Introduction

Prostate cancer encompasses a broad clinical spectrum, and treatment strategies differ substantially according to disease stage, ranging from local therapies in organ-confined disease to systemic approaches in metastatic settings [1,2]. The management of metastatic hormone-sensitive prostate cancer (mHSPC) has evolved markedly in recent years, and treatment intensification strategies have improved disease control and overall survival (OS) [2,3]. Despite these advances, the patterns of progression under contemporary first-line systemic therapy remain variable. In clinical practice, some patients experience radiologic progression limited to a small number of metastatic sites, whereas other lesions maintain stability or ongoing response to treatment [4,5].

A subset of patients receiving first-line therapy for mHSPC develops progression confined to a limited number of metastatic sites, a pattern commonly referred to as oligoprogression. Although definitions differ across studies, radiologic progression involving three or fewer lesions is widely used in clinical practice and consensus recommendations [6,7,8]. This presentation is thought to reflect focal clonal escape under an otherwise active systemic regimen, and its recognition has led to increasing interest in localized treatment strategies for carefully selected patients [9,10].

Metastasis-directed radiotherapy (RT) has been evaluated in several disease states, including hormone-sensitive settings. Trials such as STOMP and ORIOLE, which enrolled patients with hormone-sensitive oligometastatic disease, demonstrated that stereotactic RT to a limited number of lesions can delay progression and defer the need for systemic treatment intensification [11,12]. Similar concepts have been explored in castration-resistant prostate cancer (CRPC), where several retrospective analyses suggest that directing RT to isolated resistant sites may prolong the benefits of ongoing systemic therapy [13,14].

In contrast, evidence guiding the management of oligoprogression arising during first-line treatment for mHSPC remains limited. This scenario, characterized by radiologic progression restricted to only a few lesions while the remainder of the disease remains controlled, represents a relatively common yet understudied clinical problem. In practice, two main approaches are used: applying focal RT while continuing the existing systemic regimen or transitioning promptly to subsequent systemic therapy [15,16]. However, comparative data evaluating these strategies in this specific population are limited.

To address this gap, the present study evaluated outcomes in patients who developed oligoprogression while receiving first-line therapy for mHSPC and were managed either with metastasis-directed RT while continuing the same regimen or with an immediate switch to a subsequent systemic therapy.

## 2. Materials and Methods

### 2.1. Patient Selection and Study Design

This retrospective analysis included patients with metastatic hormone-sensitive prostate cancer (mHSPC) who were diagnosed and treated between 2009 and 2024. Eligibility required histologically confirmed prostate adenocarcinoma and oligoprogression during first-line systemic therapy. Oligoprogression was defined as radiologic progression in pre-existing metastatic sites, the development of new metastatic lesions, or locoregional recurrence, provided that the total number of progressing lesions was three or fewer. The diagnosis of oligoprogression was established using either conventional imaging, consisting of thoracic, abdominal, and pelvic computed tomography together with whole-body bone scintigraphy, or prostate-specific membrane antigen positron emission tomography/computed tomography (PSMA PET/CT) [17].

Patients younger than 18 years, those with concurrent active malignancies, and individuals with missing clinical or radiologic data at the time of oligoprogression were excluded. After applying these criteria, the patients were stratified into two groups according to the management strategy implemented following the first oligoprogressive event:

RT group: patients who received metastasis-directed radiotherapy to the progressive lesion(s) and continued the same systemic treatment for at least three additional months.

Treatment-change group (non-RT group): patients whose systemic therapy was switched immediately without metastasis-directed RT.

Patients who received both metastasis-directed radiotherapy and an immediate systemic treatment change were not included in the analysis.

### 2.2. Data Sources and Study Variables

Clinical, laboratory, and imaging data were retrieved from the electronic medical records and archived patient files. Demographic characteristics included age at the diagnosis of mHSPC, comorbidity status, and Eastern Cooperative Oncology Group (ECOG) performance status. Tumor-related variables comprised histological subtype, Gleason grade group, metastatic presentation, disease volume [18], and metastatic risk category [19], based on established definitions. Baseline visceral metastases, bone metastases, and the total number of bone lesions were also documented.

Laboratory values closest to the date of oligoprogression (within ±30 days) were collected, including prostate-specific antigen (PSA), hemoglobin, albumin, C-reactive protein (CRP), and serum calcium levels. Because PSA and CRP exhibited markedly right-skewed distributions with substantial interpatient variability, both variables were transformed using the natural logarithm (ln-PSA and ln-CRP) to reduce skewness, limit the influence of extreme outliers, and improve the compatibility with the assumptions of regression modeling.

Treatment-related data included the type of first-line systemic therapy administered (androgen deprivation therapy [ADT] alone, ADT plus an androgen receptor pathway inhibitor [ARPI], or ADT plus docetaxel) and the management strategy applied following oligoprogression. As described previously, patients were categorized into the RT or treatment-change group (non-RT group) based on whether metastasis-directed radiotherapy was administered at the time of oligoprogression.

In the RT group, metastasis-directed radiotherapy was delivered as linac-based stereotactic body radiotherapy after CT-based simulation and immobilization. Available imaging studies (MRI and/or PET-CT) were fused with the treatment planning CT. Gross visible lesions were contoured as the GTV (gross tumor volume). No CTV (clinical target volume) was created. PTV (planning target volume) was generated by adding a 1–3 mm margin to the GTV. Nearby organs at risk were contoured and spared according to institutional dose constraint protocols. All radiotherapy plans were made by IMRT/VMAT with 6-MV photons. A minimum of 95% PTV dose coverage was accepted. Image guidance was performed using cone-beam CT, and respiratory motion management with 4D-CT (Four-Dimensional Computed Tomography) was used for selected rib lesions.

For all patients, survival status (alive or deceased), dates of radiologic progression, initiation of subsequent systemic therapies, and date of the last follow-up visit were systematically documented. These variables formed the basis for the analysis of post-oligoprogression radiologic progression-free survival (rPFS) and overall survival (OS).

### 2.3. Definition of Survival Outcomes

Post-oligoprogression rPFS was defined from the date of oligoprogression to subsequent radiologically confirmed progression (PD2) or death, whichever occurred first. Radiologic progression was assessed using conventional imaging or PSMA PET/CT in accordance with the Response Evaluation Criteria in Solid Tumors (RECIST) or Prostate Cancer Clinical Trials Working Group 3 (PCWG3) criteria [20,21]. Patients without documented PD2 were censored on the date of their last available radiologic evaluation.

Post-oligoprogression OS was defined from the date of oligoprogression until death from any cause. For patients who remained alive, censoring was applied at the date of the most recent follow-up visit.

In the RT group, time to next systemic therapy was also evaluated and was defined as the interval between metastasis-directed radiotherapy and initiation of subsequent systemic treatment. Patients who had not started subsequent systemic therapy were censored at the date of last follow-up.

### 2.4. Ethical Considerations

This research adhered to the principles outlined in the Declaration of Helsinki. Ethical approval was granted by the Ethics Committee of Istanbul University-Cerrahpaşa, Cerrahpaşa Medical Faculty, Turkey (Approval no: 2025/302; 10 July 2025). Given the retrospective design, the requirement for informed consent was waived by the Ethics Committee in accordance with national regulations.

### 2.5. Statistical Analysis

Data analysis was performed using SPSS version 27.0 (IBM Corp., Armonk, NY, USA). The Shapiro–Wilk test was used to evaluate the normality of continuous variables. Variables that followed a normal distribution were described as mean ± standard deviation and compared using the independent samples *t*-test. Variables that did not follow a normal distribution were presented as median (minimum–maximum) and compared using the Mann–Whitney U test. Categorical variables were presented as frequencies and percentages, with group comparisons made using the chi-square test or Fisher’s exact test, as applicable. Survival analysis was conducted using the Kaplan–Meier method, and differences between groups were evaluated using the log-rank test. Patients who did not experience events were censored at their last clinical or radiological evaluation.

To determine the prognostic factors associated with rPFS and OS, univariate Cox proportional hazards regression analyses were first performed for all specified clinical, pathological, laboratory, and treatment-related variables. Variables with a univariate *p*-value of less than 0.10 were considered eligible for multivariate modeling. Because PSA and CRP levels showed a non-normal, markedly right-skewed distribution, their natural logarithmic transformations (ln-PSA and ln-CRP) were used in all regression analyses to stabilize the variance and obtain more reliable hazard ratio estimates. Multivariate Cox models were constructed using the enter method, and adjusted hazard ratios (HRs) with 95% confidence intervals (CIs) were reported.

## 3. Results

### 3.1. Baseline Clinical and Demographic Characteristics

**Figure 1:**
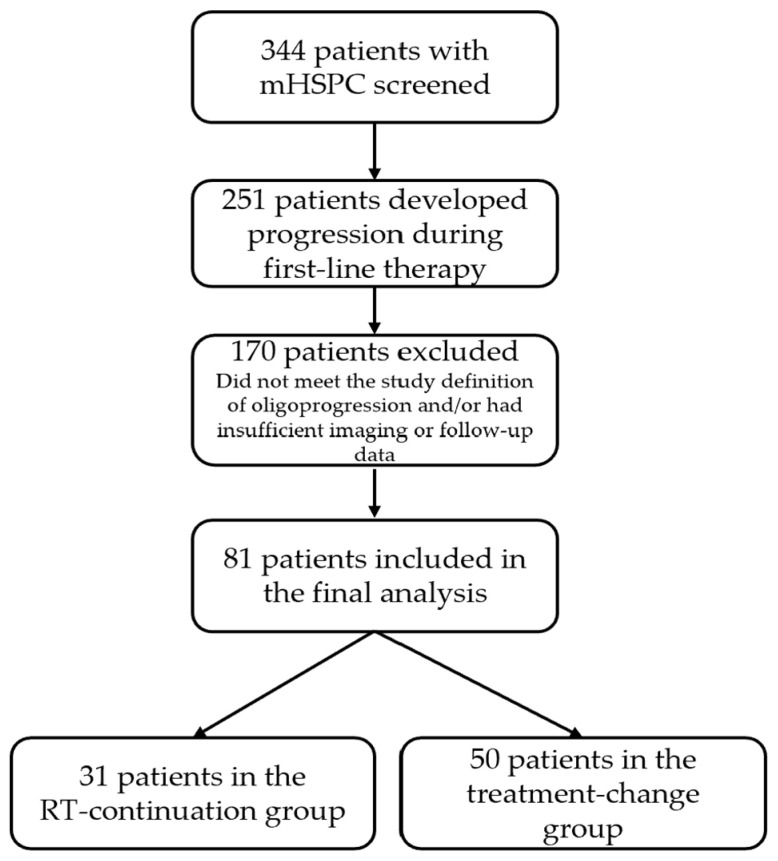
Flowchart of patient selection and study group assignment. mHSPC: metastatic hormone-sensitive prostate cancer; RT: radiotherapy.

Overall, 344 patients with mHSPC were screened, of whom 251 developed progression during first-line therapy. Among these patients, 81 (32.3%) met the study definition of oligoprogression and were included in the final analysis. Of these, 31 received metastasis-directed radiotherapy while continuing the same systemic treatment, whereas 50 underwent an immediate systemic treatment change. The process of patient selection is presented in Figure 1.

In the RT group, 31 patients received metastasis-directed radiotherapy delivered as stereotactic body radiotherapy (SBRT) for 37 lesions. Most patients were treated for a single lesion, whereas five patients received treatment for two lesions and one patient for three lesions. The irradiated targets predominantly consisted of bone lesions (34 lesions, 91.9%), whereas nodal lesions accounted for a smaller proportion (3 lesions, 8.1%). The most frequently treated sites were the vertebrae, femur, sacroiliac region, ribs, ilium, ischium, and pubic bone, whereas the nodal targets included retrocaval/renal hilar and paraaortic lymph nodes. The most commonly used dose schedules were 27 Gy in three fractions (22 patients, 71.0%) and 24 Gy in three fractions (nine patients, 29.0%). A retrospective review of the available medical records revealed no documented RT-related grade 3 or higher toxicity.

The median age at mHSPC diagnosis was 67 years (range, 42–83), and patients in the RT group were younger than those in the non-RT group (*p* = 0.044). Comorbidity burden, ECOG performance status, Gleason grade distribution, metastatic presentation, disease volume, and high-risk features were similar between the two groups. No significant differences were observed in the distribution of visceral and bone metastases or in the number of bone lesions. First-line systemic treatment patterns (ADT, ARPI, or docetaxel) were also comparable between the groups. In the overall cohort, the median time from initiation of first-line therapy to oligoprogression was 26.6 months (range, 4.1–171.9). This interval was 29.3 months (range, 4.1–171.9) in the treatment-change group and 21.4 months (range, 4.4–72.3) in the RT group, without a statistically significant difference (*p* = 0.098). The imaging modality at oligoprogression was also similar across groups, with PSMA PET/CT used in most patients in both the RT and non-RT groups (90.3% vs. 86.0%, respectively; *p* = 0.734).

Among the laboratory parameters, CRP and calcium levels at oligoprogression were higher in the RT group (*p* = 0.027 and *p* = 0.030, respectively), whereas the PSA, hemoglobin, and albumin values showed no significant differences. Table 1 summarizes the baseline demographic, clinical, and laboratory characteristics of the study cohort.

### 3.2. Post-Oligoprogression Survival Outcomes

The cohort had a median follow-up duration of 63.3 months, with a range from 15.2 to 199.7 months. During this period, radiologic progression after oligoprogression occurred in 42 of 81 patients (51.9%), and 38 of 81 patients (46.9%) died. The remaining patients were censored at their last documented clinical visit.

**Figure 2:**
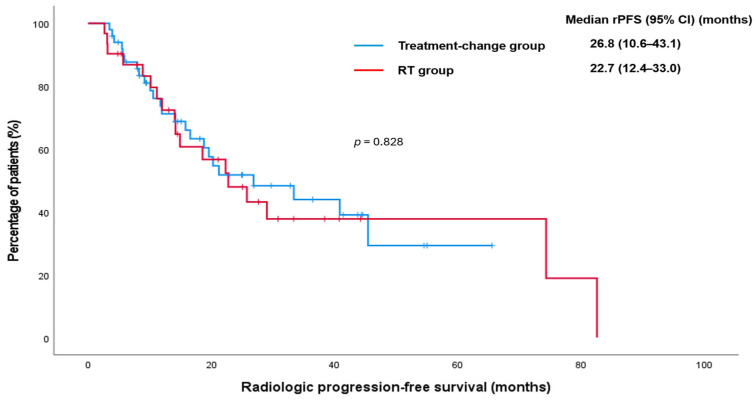
Kaplan–Meier curve for radiologic progression-free survival according to treatment strategy in patients who developed oligoprogression during first-line therapy for metastatic hormone-sensitive prostate cancer. CI: confidence interval; rPFS: radiologic progression-free survival; RT: radiotherapy.

The median post-oligoprogression rPFS for the entire cohort was 25.8 months (95% CI: 16.3–35.2). The median rPFS was 26.8 months (95% CI, 10.6–43.1) in patients who underwent an immediate change in systemic therapy and 22.7 months (95% CI, 12.4–33.0) in those who received metastasis-directed RT while continuing the same systemic regimen (*p* = 0.828) (Figure 2).

In univariate Cox analyses, poorer ECOG performance status, a higher number of bone metastases, and elevated ln-transformed CRP levels were associated with shorter rPFS (*p* = 0.047, 0.095, and 0.016, respectively). Post-oligoprogression management strategy, metastatic disease volume, metastatic risk group, and other baseline clinicopathological characteristics were not significantly associated with rPFS. In the multivariate model that included ECOG status, ln-CRP levels, and bone lesion count, none of these variables reached statistical significance, although ECOG status and ln-CRP levels demonstrated a trend toward shorter rPFS (*p* = 0.082 and *p* = 0.102, respectively). Table 2 summarizes the results of the univariate and multivariate Cox analyses for rPFS.

In addition, in the RT group, 15 of 31 patients (48.4%) initiated subsequent systemic therapy. The median time from metastasis-directed radiotherapy to the initiation of the next systemic therapy was 29.2 months. The remaining 16 patients had not started subsequent systemic treatment by their last follow-up.

**Figure 3:**
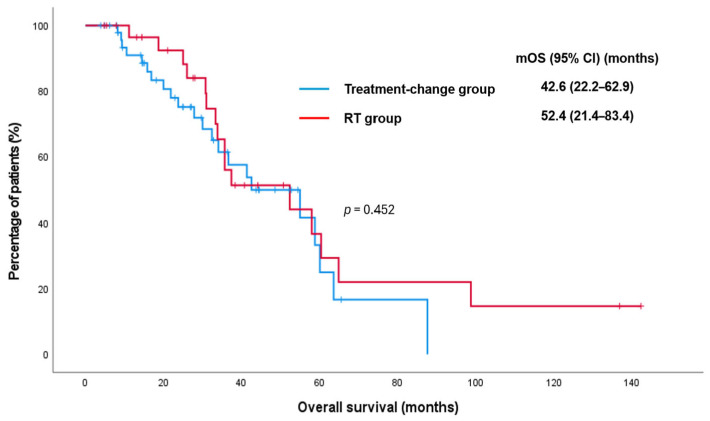
Kaplan–Meier curve for overall survival according to treatment strategy in patients who developed oligoprogression during first-line therapy for metastatic hormone-sensitive prostate cancer. CI: confidence interval; mOS: median overall survival; RT: radiotherapy.

For the entire cohort, the median OS was 42.6 months (95% CI, 24.5–60.6). According to the treatment strategy, the median OS was 42.6 months (95% CI, 22.2–62.9) in patients who underwent an immediate systemic treatment change and 52.4 months (95% CI, 21.4–83.4) in those who received metastasis-directed RT while continuing the same systemic regimen (*p* = 0.452) (Figure 3).

In univariate Cox analyses, older age, poorer ECOG performance status, lower hemoglobin levels, and lower albumin levels were associated with shorter OS (*p* = 0.004, 0.048, 0.023, and 0.028, respectively). The metastatic risk group demonstrated borderline significance (*p* = 0.052). Other baseline clinical and laboratory factors, including post-oligoprogression management strategy, type of metastatic presentation, metastatic volume, visceral or bone metastases, bone lesion count, first-line systemic treatment, and ln-PSA, did not show a significant association with OS.

In the multivariate model that included age, ECOG status, metastatic risk group, hemoglobin, and albumin, none of these variables reached statistical significance, although age and metastatic risk groups demonstrated a trend toward shorter OS (*p* = 0.064 and *p* = 0.072, respectively). Table 3 summarizes the findings from the univariate and multivariate Cox analyses for OS.

## 4. Discussion

In our retrospective cohort, post-oligoprogression rPFS and OS did not differ significantly between patients managed with metastasis-directed RT while continuing the same regimen and those who underwent an immediate switch to subsequent systemic therapy. Although median OS was numerically longer in the RT group, this difference did not reach statistical significance. Given the limited data specifically addressing oligoprogression that occurs during first-line treatment for mHSPC and the absence of well-established comparative evidence for these two management strategies in this setting, the results should be interpreted within the context of the currently limited evidence base.

Metastasis-directed therapy has been evaluated in several clinical settings, including the metastatic hormone-sensitive disease state. The STOMP and ORIOLE randomized phase II trials, in which stereotactic RT was compared with observation rather than systemic treatment escalation, demonstrated that treating a limited number of metastatic sites can delay disease progression and defer the initiation of systemic therapy [11,12]. Although these studies provide a rationale for targeting focal lesions, the patient populations enrolled—individuals with recurrent hormone-sensitive prostate cancer and a limited metastatic burden who were not receiving ongoing systemic therapy—differ substantially from patients who develop oligoprogression during active first-line treatment for mHSPC.

In the castration-resistant setting, several retrospective studies have evaluated metastasis-directed RT for oligoprogressive diseases. Ingrosso et al. analyzed 34 patients with mCRPC receiving androgen receptor–targeted therapy and reported that adding SBRT to sites of oligoprogression prolonged the efficacy of ongoing systemic treatment and favorably influenced disease progression [10]. In a separate cohort, Triggiani et al. demonstrated that stereotactic RT for a limited number of progressing metastases was associated with an extended duration of the same systemic therapy and improvements in progression-related outcomes [14]. A comprehensive review further emphasized that ablating resistant lesions may help suppress emerging clones and maintain sensitivity to active systemic agents in patients with oligoprogressive CRPC [22].

In our RT group, the median time from metastasis-directed radiotherapy to initiation of subsequent systemic therapy was 29.2 months, suggesting that, in selected patients, local treatment may help maintain the same systemic regimen for a clinically meaningful period. Among the available evidence, the study by Deek et al. is particularly relevant to our conceptual framework. In their cohort of patients with oligoprogressive mCRPC, outcomes were compared between those who continued the same systemic treatment with the addition of metastasis-directed therapy (MDT) and those who underwent a treatment change. MDT was associated with improved time to next intervention (TTNI) and distant metastasis–free survival (DMFS), supporting the potential role of focal therapy in extending systemic treatment benefit even in a more treatment-resistant context [9]. Similarly, Eule et al., in a study of 32 patients with oligoprogressive mCRPC, reported a median time to next systemic therapy of 10.1 months after SBRT [23]. These observations highlight the biological rationale underlying MDT; however, their applicability to patients who develop oligoprogression during first-line therapy for mHSPC, where disease biology and prior treatment exposure differ substantially from that of CRPC, remains uncertain. Most prior studies have focused on oligoprogressive mCRPC and have included patients treated at different stages of systemic therapy, usually in retrospective cohorts evaluating MDT [9,10,23,24]. In our study, by contrast, we specifically examined patients who developed oligoprogression during first-line systemic treatment for mHSPC and directly compared two management strategies in this setting. However, our findings should be interpreted with caution, as the number of events in the RT group in our study was limited.

The lack of a significant difference between metastasis-directed RT and systemic treatment change in our cohort may reflect the intrinsic features of oligoprogression that emerge during first-line therapy for mHSPC. In patients who experience limited progression while otherwise responding to initial systemic treatment, both focal RT and a switch to subsequent systemic therapy may be considered in clinical practice. Within this context, although our findings do not support an interpretation of equivalence between the two strategies, they suggest that more than one management approach may be considered in selected patients.

Our findings reflect routine clinical practice in patients with oligoprogression during first-line treatment for mHSPC. However, the results should be interpreted in light of the following limitations. Given the retrospective study design, bias related to patient selection cannot be ruled out, and the relatively small cohort restricts the reliability of the subgroup analyses. Treatment allocation was not randomized and was based on clinical judgment, introducing the possibility of confounding by indication. Differences between the groups, including age, histopathology, and CRP and calcium levels at oligoprogression, may have contributed to the observed outcomes. In particular, the younger age distribution in the RT group may have influenced the numerically longer overall survival observed in that group despite the absence of a statistically significant difference. Although multivariable analyses were performed, residual confounding cannot be excluded. Another limitation of this study is the heterogeneity of imaging modalities used to define oligoprogression. Most patients were assessed with PSMA PET/CT, whereas a minority were evaluated with conventional imaging. Given the higher sensitivity of PSMA PET/CT for lesion detection, this variability may have influenced the detection and characterization of progressing lesions and the classification of oligoprogression. Although all metastasis-directed radiotherapy in this cohort consisted of SBRT, changes in radiotherapy techniques and delivery approaches over the long study period may have influenced the consistency of treatment delivery and the observed outcomes. In addition, because of the retrospective design, detailed lesion-level radiotherapy parameters, lower-grade toxicity data, and local control of irradiated lesions were not uniformly captured across all patients. Finally, a notable proportion of patients received ADT alone as the initial systemic therapy, reflecting the treatment practices of the time but potentially limiting the generalizability of the findings to contemporary management patterns.

## 5. Conclusions

In patients with mHSPC who developed oligoprogression during first-line systemic therapy, metastasis-directed RT with continuation of the same regimen and immediate change in systemic treatment were associated with comparable post-oligoprogression outcomes in our cohort. These findings suggest that both strategies may represent feasible management approaches in selected patients. However, given the observational nature of this study, further prospective studies using standardized imaging and contemporary treatment approaches, including both systemic therapy and radiotherapy, would help clarify which patient subgroups may benefit more from each strategy.

## Figures and Tables

**Table 1:** Baseline characteristics of the study population according to the management strategy used after oligoprogression.

Variables		Overall Cohort (*n* = 81)	RT Group (*n* = 31)	Non-RT Group (*n* = 50)	*p* Value
Age at mHSPC diagnosis, years	Median (Range)	67 (42–83)	60 (42–80)	68 (50–83)	0.044
	≥65, *n* (%)	45 (55.6)	14 (45.2)	31 (62)	0.138
Comorbidity *n* (%)	Absent Present	31 (38.3) 50 (61.7)	10 (32.3) 21 (67.7)	21 (42) 29 (58)	0.381
ECOG PS *n* (%)	0 ≥1	43 (53.1) 38 (46.9)	16 (51.6) 15 (48.4)	27 (54) 23 (46)	0.334
Gleason Grade Group *n* (%)	1–2 3 4–5	12 (14.8) 16 (19.8) 53 (65.4)	4 (12.9) 6 (19.4) 21 (67.7)	8 (16) 10 (20) 32 (64)	0.919
Histopathology *n* (%)	Acinar Ductal Mixed	71 (87.6) 5 (6.2) 5 (6.2)	27 (87.1) 4 (12.9) 0 (0)	44 (88) 1 (2) 5 (10)	0.034
Type of metastatic disease, *n* (%)	Metachronous Synchronous	30 (37) 51 (63)	9 (29) 22 (71)	21 (42) 29 (58)	0.240
Metastatic volume *n* (%)	High-volume Low-volume	32 (39.5) 49 (60.5)	14 (45.2) 17 (54.8)	18 (36) 32 (64)	0.412
Metastatic risk *n* (%)	High-risk Low-risk	29 (35.8) 52 (64.2)	14 (45.2) 17 (54.8)	15 (30) 35 (70)	0.167
Visceral metastasis *n* (%)	Absent Present	76 (93.8) 5 (6.2)	28 (90.3) 3 (9.7)	48 (96) 2 (4.0)	0.302
Bone metastasis *n* (%)	Absent Present	13 (16) 68 (84)	3 (9.7) 28 (90.3)	10 (20) 40 (80)	0.219
Bone lesion count *n* (%)	1–3 ≥4	32 (47.1) 36 (52.9)	13 (46.4) 15 (53.6)	19 (47.5) 21 (52.5)	0.931
First-line therapy *n* (%)	Only ADT ADT + ARPI ADT + Docetaxel	37 (45.7) 20 (24.7) 24 (29.6)	14 (45.2) 7 (22.6) 10 (32.3)	23 (46) 13 (26) 14 (28)	0.900
Time to oligoprogression, months	Median (Range)	26.6 (4.1–171.9)	21.4 (4.4–72.3)	29.3 (4.1–171.9)	0.098
Imaging modality at oligoprogression, *n* (%)	Conventional imaging PSMA PET/CT	10 (12.3) 71 (87.7)	3 (9.7) 28 (90.3)	7 (14) 43 (86)	0.734 *
PSA at oligoprogression (ng/mL)	Median (Range)	50 (1.22–3755)	54 (1.72–1700)	46 (1.22–3755)	0.954
Hemoglobin at oligoprogression (g/dL)	Median (Range)	13.0 (7.8–17.2)	13.1 (7.8–17.2)	13.0 (9.3–14.9)	0.397
Albumin at oligoprogression (g/dL)	Median (Range)	4.0 (3.3–10.4)	4.37 (3.4–10.4)	4.0 (3.3–4.98)	0.915
CRP at oligoprogression (mg/L)	Median (Range)	2.5 (0.38–123)	3.87 (0.40–123)	2.50 (0.38–24)	0.027
Calcium at oligoprogression (mg/dL)	Median (Range)	9.3 (2.2–10.5)	9.6 (8.3–10.5)	9.4 (2.2–10.5)	0.030

* For imaging modality, the p-value was calculated using Fisher’s exact test. ADT: androgen deprivation therapy; ARPI: androgen receptor pathway inhibitor; CRP: C-reactive protein; ECOG PS: Eastern Cooperative Oncology Group performance status; mHSPC: metastatic hormone-sensitive prostate cancer; PSA: prostate-specific antigen; PSMA PET/CT: prostate-specific membrane antigen positron emission tomography/computed tomography.

**Table 2:** Univariate and multivariate Cox model results for post-oligoprogression rPFS.

	Univariate Analyses		Multivariate Analyses	
Variable	HR (95% CI)	*p*-Value	HR (95% CI)	*p*-Value
Age (≥65 vs. <65 years)	1.09 (0.58–2.04)	0.793		
Comorbidity (Present vs. absent)	1.26 (0.66–2.43)	0.488		
ECOG PS (≥1 vs. 0)	1.80 (1.01–3.21)	0.047	1.80 (0.93–3.49)	0.082
Gleason grade group (1–2 vs. 3 vs. 4–5)	0.90 (0.62–1.30)	0.573		
Histopathology	0.99 (0.56–1.76)	0.975		
Type of metastatic disease (Synchronous vs. metachronous)	1.20 (0.63–2.28)	0.585		
Metastatic volume (Low vs. High)	0.71 (0.38–1.32)	0.276		
Metastatic risk (Low-risk vs. High-risk)	0.64 (0.33–1.24)	0.187		
Visceral metastasis (Absent vs. Present)	0.39 (0.05–3.05)	0.368		
Bone metastasis (Absent vs. Present)	1.02 (0.45–2.32)	0.957		
Bone lesion count (≥4 vs. 1–3)	1.80 (0.90–3.58)	0.095	1.61 (0.79–3.27)	0.192
Post-oligoprogression management strategy (RT-continuation vs. treatment change)	1.07 (0.57–2.02)	0.828		
First-line therapy (ADT vs. ARPI vs. Docetaxel)	0.94 (0.75–1.17)	0.583		
ln-PSA (Per 1-unit increase)	1.09 (0.90–1.31)	0.379		
Hemoglobin (g/dL) (Per 1 g/dL increase)	0.87 (0.71–1.07)	0.199		
Albumin (g/dL) (Per 1 g/dL increase)	1.29 (0.85–1.95)	0.226		
Calcium (mg/dL) (Per 1 mg/dL increase)	0.90 (0.73–1.11)	0.329		
ln-CRP (Per 1-unit increase)	1.39 (1.06–1.81)	0.016	1.27 (0.95–1.68)	0.102

CI: confidence interval; CRP: C-reactive protein; ECOG PS: Eastern Cooperative Oncology Group performance status; HR: hazard ratio; PSA: prostate-specific antigen; RT: radiotherapy.

**Table 3:** Univariate and multivariate Cox model results for post-oligoprogression OS.

	Univariate Analyses		Multivariate Analyses	
Variable	HR (95% CI)	*p*-Value	HR (95% CI)	*p*-Value
Age (≥65 vs. <65 years)	2.74 (1.38–5.43)	0.004	2.29 (0.95–5.48)	0.064
Comorbidity (Present vs. absent)	0.75 (0.38–1.48)	0.412		
ECOG PS (≥1 vs. 0)	1.84 (1.01–3.38)	0.048	1.25 (0.64–2.45)	0.513
Gleason grade group (1–2 vs. 3 vs. 4–5)	1.06 (0.71–1.59)	0.774		
Histopathology	0.92 (0.50–1.71)	0.793		
Type of metastatic disease (Synchronous vs. metachronous)	0.98 (0.51–1.86)	0.941		
Metastatic volume (Low vs. High)	0.76 (0.40–1.45)	0.406		
Metastatic risk (Low-risk vs. High-risk)	0.52 (0.27–1.00)	0.052	0.52 (0.25–1.06)	0.072
Visceral metastasis (Absent vs. Present)	0.80 (0.18–3.47)	0.763		
Bone metastasis (Absent vs. Present)	1.40 (0.59–3.36)	0.449		
Bone lesion count (≥4 vs. 1–3)	1.59 (0.78–3.25)	0.199		
Post-oligoprogression management strategy (RT-continuation vs. treatment change)	0.78 (0.40–1.51)	0.454		
First-line therapy (ADT vs. ARPI vs. Docetaxel)	0.95 (0.76–1.19)	0.659		
ln-PSA (Per 1-unit increase)	1.07 (0.89–1.29)	0.443		
Hemoglobin (g/dL) (Per 1 g/dL increase)	0.76 (0.59–0.96)	0.023	0.94 (0.70–1.26)	0.666
Albumin (g/dL) (Per 1 g/dL increase)	0.41 (0.19–0.91)	0.028	0.73 (0.30–1.77)	0.488
Calcium (mg/dL) (Per 1 mg/dL increase)	0.97 (0.79–1.19)	0.779		
ln-CRP (Per 1-unit increase)	1.23 (0.93–1.62)	0.151		

CI, confidence interval; CRP, C-reactive protein; ECOG PS, Eastern Cooperative Oncology Group performance status; HR, hazard ratio; PSA, prostate-specific antigen; RT, radiotherapy.

## Data Availability

The data underlying this study are available from the corresponding author on reasonable request.

## Abbreviations

The following abbreviations are used in this manuscript:

ADT	Androgen Deprivation Therapy
ARPI	Androgen Receptor Pathway Inhibitor
CI	Confidence Interval
CRP	C-Reactive Protein
CRPC	Castration-Resistant Prostate Cancer
CTV	Clinical Target Volume
ECOG PS	Eastern Cooperative Oncology Group Performance Status
GTV	Gross Tumor Volume
HR	Hazard Ratio
IMRT	Intensity-Modulated Radiotherapy
ln-CRP	Natural Logarithm of C-Reactive Protein
ln-PSA	Natural Logarithm of Prostate-Specific Antigen
mCRPC	Metastatic Castration-Resistant Prostate Cancer
MDT	Metastasis-Directed Therapy
mHSPC	Metastatic Hormone-Sensitive Prostate Cancer
mOS	Median Overall Survival
MRI	Magnetic Resonance Imaging
OS	Overall Survival
PCWG3	Prostate Cancer Clinical Trials Working Group 3
PD2	Second Radiologic Progression
PET/CT	Positron Emission Tomography/Computed Tomography
PSA	Prostate-Specific Antigen
PSMA	Prostate-Specific Membrane Antigen
PTV	Planning Target Volume
RECIST	Response Evaluation Criteria in Solid Tumors
rPFS	Radiological Progression-Free Survival
RT	Radiotherapy
SBRT	Stereotactic Body Radiotherapy
TTNI	Time to Next Intervention
VMAT	Volumetric Modulated Arc Therapy
4D-CT	Four-Dimensional Computed Tomography
